# Therapeutic effect of F127-folate@PLGA/CHL/IR780 nanoparticles on folate receptor-expressing cancer cells

**DOI:** 10.3762/bjnano.15.78

**Published:** 2024-07-31

**Authors:** Thi Ngoc Han Pham, Phuong-Thao Dang-Luong, Hong-Phuc Nguyen, Loc Le-Tuan, Xuan Thang Cao, Thanh-Danh Nguyen, Vy Tran Anh, Hieu Vu_Quang

**Affiliations:** 1 NTT Hi-tech institute, Nguyen Tat Thanh University, Ho Chi Minh City, 700000, Vietnam; 2 Faculty of Chemical Engineering, Industrial University of Ho Chi Minh City, Ho Chi Minh City, 700000, Vietnam; 3 Institute of Chemical Technology – VAST, Ho Chi Minh City, 700000, Vietnam; 4 Institute of Applied Technology and Sustainable Development, Nguyen Tat Thanh University, Ho Chi Minh City 700000, Vietnam

**Keywords:** cancer, chlorambucil, drug carrier, IR780, PLGA nanoparticle, theragnostic

## Abstract

Theragnostic platforms, which integrate therapeutic and diagnostic capabilities, have gained significant interest in drug research because of to their potential advantages. This study reports the development of a novel multifunctional nanoparticle carrier system based on poly(ᴅ,ʟ-lactic-*co*-glycolic acid) (PLGA) for the targeted delivery of the chemotherapeutic agent chlorambucil (CHL) and the imaging agent IR780. The approach in this study incorporates Pluronic F127-folate onto the PLGA nanoparticles, which enables targeted delivery to folate receptor-expressing cancer cells. The F127-folate@PLGA/CHL/IR780 nanoparticles were formulated using a nanoprecipitation technique, resulting in small size, high homogeneity, and negative surface charge. Importantly, the folate-targeted nanoparticles demonstrated enhanced uptake and cytotoxicity in folate receptor-positive cancer cell lines (MCF-7 and HepG-2) compared to folate receptor-negative cells (HEK 293). Additionally, the F127-folate@PLGA/CHL/IR780 nanoparticles exhibited a lower IC_50_ value against cancer cells than non-targeted F127@PLGA/CHL/IR780 nanoparticles. These findings suggest that the developed F127-folate@PLGA/CHL/IR780 nanoparticles hold promise as a theragnostic system for targeted cancer therapy and diagnosis, leveraging the advantages of PLGA, folate targeting, and the integration of therapeutic and imaging agents.

## Introduction

Poly(ᴅ,ʟ-lactic-*co*-glycolic acid) (PLGA), a copolymer of poly(lactic acid) (PLA) and poly(glycolic acid) (PGA), has been employed in the synthesis of nanoparticles for cancer diagnostics and therapy because of its excellent biocompatibility and biodegradability [1–3]. PLGA nanoparticles can be prepared via a variety of techniques, including single emulsion, double emulsion, or nanoprecipitation [1,4], in which copolymers are dissolved in an organic solvent, called the organic phase, and then are put into an immiscible aqueous solution, called the water phase, to form the nanoparticles. Various surfactants, including poly(vinyl alcohol) (PVA), sodium cholate, or pluronic F127 (F127), can be used in the water phase to lower the surface tension of the organic phase and to produce the nanoemulsion during the homogenization process [5–7]. F127 is a copolymer made up of blocks of poly(ethylene oxide)–poly(propylene oxide)–poly(ethylene oxide), PEO100–PPO65–PEO100. The outer PEO corona prevents aggregation, protein adsorption, and recognition by the reticuloendothelial system, while the hydrophobic PPO core may be modified to contain hydrophobic anticancer drugs, fluorophores, or even anchor to the hydrophobic layer of the nanoparticles [8–9].

The F127 copolymer has been utilized in many studies to develop a carrier that is effective for both treatment and diagnostics [8–9]. Its delivery efficiency can be improved by modifying the substance with targeted ligands such as folic acid, RGD, or antibodies [10–11]. Folic acid is one of the most common ligands used. Its receptor is significantly overexpressed in several types of cancer cells, while there is an undetectable expression in normal cells [12]. Hence, the incorporation of folic acid into nanoparticles is helpful in actively targeting tumors [12–13]. In our previous study, F127 was conjugated with folic acid to enhance the imaging contrast in magnetic resonance imaging (MRI) or to improve the therapeutic efficacy of nanoparticles [14].

Chlorambucil (CHL) is a nitrogen mustard alkylating drug used to treat several benign tumors and malignancies, including chronic lymphatic leukemia [15], Hodgkin’s and non-lymphoma Hodgkin’s [16–17], advanced ovarian and breast cancer [17–18], and some autoimmune illnesses [19]. Like other alkylating drugs, CHL inhibits tumor growth by cross-linking guanine or adenine bases in DNA double helix strands, preventing them from uncoiling and separating, and consequently disrupting DNA replication in proliferating cancer cells. The neurotoxicity and myelotoxicity of CHL, however, limit its clinical use [20–21]. Moreover, the instability of chemotherapeutics due to the hydrolysis of the chloroethyl group significantly hampers their therapeutic impact [22]. Therefore, it is essential to prevent CHL hydrolysis by incorporating it into nanoparticles, boosting its stability.

IR780 iodide is a lipophilic heptamethine NIR fluorophore that absorbs light with a peak at 780 nm. It turns NIR (750–1000 nm) laser energy into heat after being exposed to it. It was discovered to have exceptional inherent tumor-targeting characteristics without any modification, and it is a fluorophore enabling near-infrared imaging. However, IR780 iodide has low water stability and photostability [23] and shows acute toxicity at high doses [24], which limits its clinical application. To address these issues, many studies have attempted to encapsulate IR780 iodide in different types of nanoparticles (NPs) [23–25] to make use of the protective cover provided by the NPs. In addition, IR780-loaded NPs have been proven to inhibit lung metastasis when exposed to a NIR laser [24].

It has been found that PLGA may co-encapsulate a range of hydrophobic and hydrophilic compounds, which, because of the core–shell structures, boosts their therapeutic anti-tumor activity [1–3,11,26–33]. In this study, we would want to combine all of the advantages of CHL, IR780, and PLGA nanoparticles in order to create a carrier that could deliver the drug, track the location of the tumor, and control the release of the drug. The nanoparticles would increase the drug’s biocompatibility and stability. In addition, the nanoparticles were modified with the targeting ligand folate to improve their capacity to target cells expressing the folate receptor.

## Materials and Methods

Chlorambucil (C0253), PLGA 504H (719900), IR780 (425311), Pluronic™ F-127 (P2443), poly(vinyl alcohol) (PVA) (P8136), phosphate buffered saline (PBS) (P4417), dimethyl sulfoxide (DMSO) (472301), dichloromethane (DCM), 3-[4,5-dimethylthiazol-2-yl]-2,5-diphenyl-2*H*-tetrazolium bromide (MTT) (M2128), coumarin-6 (Cou-6) (442631), folic acid (1039840005) and 1,1-carbonyldiimidazole (115533) were purchased from Sigma-Aldrich. Dulbecco’s Modified Eagle Medium (DMEM) (11965092), fetal bovine serum (FBS) (MT35010CV), antibiotic (15-240-062), and trypsin (25-200-056) were purchased from Gibco, Fisher Scientific.

### Conjugation of pluronic F127 and folic acid

The conjugation of folic acid to Pluronic F127 was done as described in our previous study [34]. Folic acid (0.4 mmol) was activated by 1,1-carbonyldiimidazole (CDI) (0.44 mmol) in 6 mL of dry dimethyl sulfoxide (DMSO) in a dark environment for 24 h. The mixture was agitated for another 24 h at room temperature after the addition of F127 (0.1 mmol). The reaction was then diluted with 50% distilled water and dialyzed (MWCO 3500 Dal) against deionized water for three days (the water was changed twice a day). The solution was then freeze-dried for three days and stored at −80 °C until usage. The final product was confirmed by NMR analysis (Supporting Information File 1, Figure S1).

### Formulation of F127-folate@PLGA/CHL/IR780 nanoparticles

In acetonitrile, PLGA 504H (20 mg), chlorambucil (2 mg), and IR780 (0.1 mg) were mixed (2 mL). Then, the mixture was added dropwise into 20 mL of F127 solution (1.5% w/v) with F127-folate (2 mg) while stirring at 200 rpm. For 16 h, the organic solvent was allowed to evaporate. The F127-folate@PLGA/CHL/IR780 were then collected by 30 min of centrifugation at 12,000 rpm.

Another set of particles, such as F127@PLGA/Cou-6, and F127-folate@PLGA/Cou-6, were used in the cell targeting experiment, which were prepared under similar conditions.

### Characterization of F127-folate@PLGA/CHL/IR780 nanoparticles

Dynamic light scattering (DLS) and zeta potential spectra measurements were carried out in three replicates on a nanoPartica Horiba SZ-100 (Japan) with a scattering angle of 90° at 25 °C to determine the size distribution and stability of the nanocomposites.

### Scanning electron microscopy (SEM)

The F127-folate@PLGA/CHL/IR780 (10 μL) suspension was loaded on a silica film for 1 min, and water was allowed to evaporate. Then, the nanoparticles were coated with titanium, and the SEM images were acquired using a FE-SEM S4800 HITACHI, Japan.

### Drug loading efficiency

The F127-folate@PLGA/CHL/IR780 suspension was freeze-dried and weighed in small quantities. After dissolving the powder in acetonitrile (20 μL), it was precipitated in methanol (100 μL). Using a NanoDrop One^C^ apparatus (Thermal Scientific), the drug concentration in F127-folate@PLGA/CHL/IR780 was measured at 258 nm for CHL and 780 nm for IR780. Then, the drug content in the formulation was determined according to


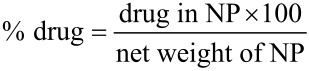


### Drug release from nanoparticles

The F127-folate@PLGA/CHL/IR780 were kept in diluted 0.1× PBS (NaCl 13.7 mM, KCl 0.27 mM, NaH_2_PO_4_ 1 mM, and KHPO_4_ 0.18 mM) and incubated at 37 °C for various time points, (24, 48, 72, and 168 h) at 37 °C at pH 7.4 and pH 5.4. After that, the nanoparticles were centrifuged at 12,000 rpm, and the pellets were collected and freeze-dried. The drugs that remained in the nanoparticle were determined as described above.

### Cell culture

The human breast carcinoma MCF7 cell line, the human hepatoma HepG2 cell line, and the human embryonic kidney HEK 293 cell line were grown in DMEM (Gibco), 10% fetal bovine serum (FBS) (Gibco), 1% antibiotics, and 5% CO_2_ at 37 °C. One well of the 96-well plate was seeded with 5000 cells for the MTT experiment, with four replicates per test.

### Targeting evaluation

The F127-folate@PLGA/Cou-6 and F127@PLGA/Cou-6 were used to evaluate the targeting ability of the nanoparticles. Before the experiment, the cells were seeded in 24-well plates at a density of 50,000 cells per well and incubated overnight. The next day, a few wells were pretreated with folic acid (2 μM) and F127 (2 mg/mL) separately for 30 min. Then, the cells were incubated for 3 h with different nanoparticles (0.1 μg/mL), which included F127-folate@PLGA/Cou-6, F127@PLGA/Cou-6, F127-folate@PLGA/Cou-6, folic acid, and F127@PLGA/Cou-6 and F127. The cells were then washed three times with PBS, collected, counted, and distributed (20.000 cells per well) to a 96-well black plate. The cells without treatment were used as a control. The Cou-6 signal from the cells was read by the VICTOR Nivo Multimode Microplate Reader, Pelkin Elmer, USA, at wavelengths of excitation and emission at 450 and 510 nm, respectively.

### In vitro cytotoxicity

The toxicity of F127-folate@PLGA/CHL/IR780 was assessed by the MTT test. Cells were treated with these nanoparticles at several concentrations (0.5, 1, and 1.5 mg/mL) and time points (24, 48, and 72 h) and then assessed. The cells were then exposed to MTT for 4 h. The medium was removed with care, and the crystal violet precipitate was dissolved with DMSO. The samples were then measured at 562 nm. The untreated cell was employed as a negative control. The cells treated with CHL served as a positive control.

## Results and Discussion

Controlled release, biocompatibility, targeted distribution, decreased toxicity, and versatility are some of the benefits of PLGA nanoparticles [1,3–4]. Therefore, this study aimed to synthesize PLGA nanoparticles with these advantages. The PLGA nanoparticles in this study were designed to contain CHL, a well-known cancer medication [19], as well as IR780, a fluorescent dye used to visualize particle location using infrared light [25]. Researchers have also found that F127-folate helps the nanoparticle to penetrate cancer cells through the folate receptor. This makes the treatment work better [12–14,34–37]. Our nanoparticles were also modified with F127-folate to enhance their therapeutic efficacy.

### Size and charge of the nanoparticles

**Figure 1:**
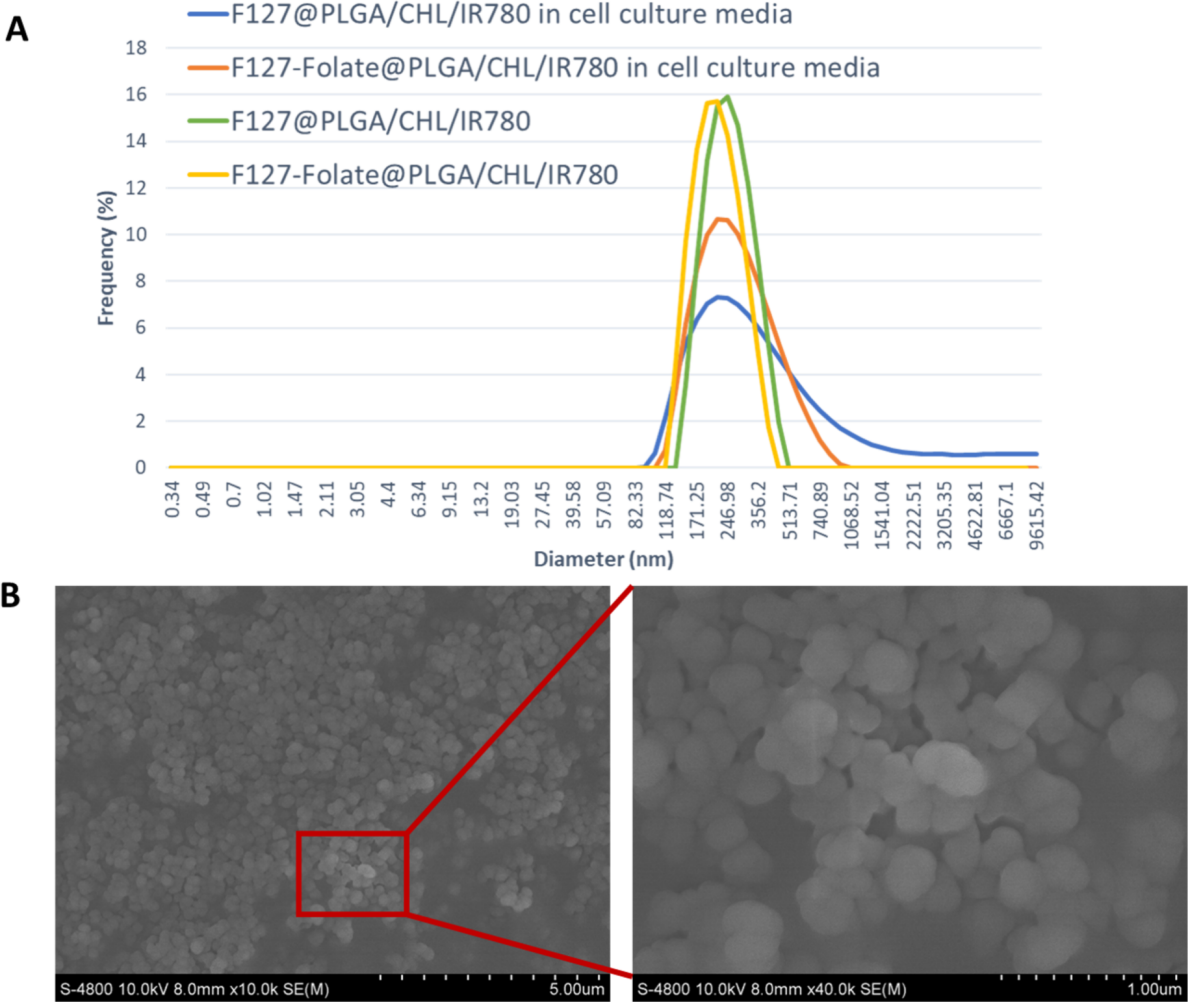
(A) Hydrodynamic size of F127-folate@PLGA/CHL/IR780 and F127@PLGA/CHL/IR780 in 0.1× PBS and in cell culture medium and (B) SEM image of F127-folate@PLGA/CHL/IR780 at magnifications of 10k and 40k.

PLGA nanoparticles can be synthesized by several methods, such as single emulsion evaporation, double emulsion evaporation, or microfluidics using different surfactants, including PVA, F127, sodium cholate, or SDS [1–5]. In the study, we employed two methods, namely, single emulsion–evaporation and nanoprecipitation–diffusion, using F127 as a surfactant. The nanoparticles formulated by the emulsion–evaporation approach were not uniform and tended to aggregate (data not shown), while the nanoparticles generated by the nanoprecipitation–diffusion method were homogeneous with a polydispersity index (PDI) of less than 0.075 ± 0.05 and a size of 198 ± 5 nm for F127-folate@PLGA/CHL/IR780 and 228 ± 4 nm for F127@PLGA/CHL/IR780 (Table 1 and Figure 1A). SEM images showed that the nanoparticles have a spherical form with a core size of around 100 nm (Figure 1B). For this reason, the nanoprecipitation–diffusion technique was utilized to produce the particles employed in the study.

**Table 1:** The hydrodynamic size and zeta potential of the F127-folate@PLGA/CHL/IR780 and F127@PLGA/CHL/IR780, (*n* = 3).

	Size (nm) Z-average	Polydispersity index (PDI)	Zeta potential mV	Chlorambucil loading	IR780 loading

F127-folate@PLGA/CHL/IR780	198 ± 5	0.075 ± 0.05	−84.3 ± 2.5	0.5% ± 0.1%	0.26% ± 0.1%
F127@PLGA/CHL/IR780	228 ± 4	0.072 ± 0.02	−77.4 ± 3		
F127-folate@PLGA/CHL/IR780 in cell culture media	371.5 ± 54	0.486 ± 0.12			
F127@PLGA/CHL/IR780 in cell culture media	287.7 ± 74	0.663 ± 0.15			

Furthermore, after dispersing the nanoparticles in cell culture medium with 10% FBS, the size and PDI of the nanoparticles increased, respectively, to 372 nm and 0.486 for F127-folate@PLGA/CHL/IR780, and, respectively, to 288 nm and 0.663 for F127@PLGA/CHL/IR780. The increase in nanoparticle size could be the result of protein absorption the nanoparticle surface [38]. However, the absorption of protein from the cell culture medium did not affect the dispersion of nanoparticles; both F127-folate@PLGA/CHL/IR780 and F127@PLGA/CHL/IR780 did not aggregate much and remained well dispersed.

Many factors contribute to an effective treatment using nanoparticles [39]. Among them, two can be deemed essential, namely, the half-life of the nanoparticles and the targeting ligands. Longer retention times for nanoparticles make it more likely that they will target the right area with the right targeting ligands, which leads to more nanoparticles building up in cancer cells.

Nanoparticle size, charge, and composition all have an impact on the half-life of nanoparticles that are currently in circulation. The optimal nanoparticle size for cancer therapy was observed to vary between 50 and 200 nm [39–40]. In our study, the nanoparticles were tailored for in vivo drug administration; thus, our nanoparticle size was suitable for use in cancer treatment. The bigger the nanoparticles, the faster they would be cleared by the mononuclear phagocyte system [41], while those smaller than 6 nm would be excreted into the Bowman space of the kidney [42].

In addition to size, nanoparticle charge is also crucial for boosting its circulation in the system. A zero charge or a slightly negative charge on the nanoparticles would prevent them from aggregating and interacting with blood proteins [43]. Our nanoparticles’s zeta potential in ten-time diluted PBS was −84.3 ± 2.5 mV and −77.4 ± 3 mV for F127-folate@PLGA/CHL/IR780 and F127@PLGA/CHL/IR780, respectively (Table 1), which is acceptable for systemic administration. According to a review, these negative charges could reduce kidney excretion. The endothelial glycocalyx layer, glomerular basement membrane, and podocyte glycocalyx layer have negative charges, which filter positive nanoparticles faster than the negative ones [42].

Preventing protein absorption by nanoparticles might enhance the systemic half-life of nanoparticles. According to reports, nanoparticles with a stealth surface have a longer half-life [39]. Typically, hydrophilic substances, such as PVA and PEG, serve as stealth surface materials. The polymers used in our study were made of pluronic F127. The polymers consist of three polymer blocks, including two PEO blocks and one PPO block. Because of the presence of F127 on the particles’ surface, the nanoparticles were effectively disseminated in our study. The hydrophobic block PPO of F127 associates with or anchors to hydrophobic PLGA, while the hydrophilic block PEO exposed to the aqueous environment associates with or anchors to hydrophilic PLGA [9–10]. The PEO block yields a stealthy surface that inhibits protein adsorption and aggregation. In addition, the conjugation of folate to the terminal PEO block increased the targeting efficiency. The externalization of the PEO chain would enhance the likelihood of folate binding to the overexpressed folate receptor on the surface of cancer cells. The difference in zeta potential between F127-folate@PLGA/CHL/IR780 and F127@PLGA/CHL/IR780 could be due to the presence of F127-folate on the surface of the nanoparticles (Table 1).

### Targeting ligand

**Figure 2:**
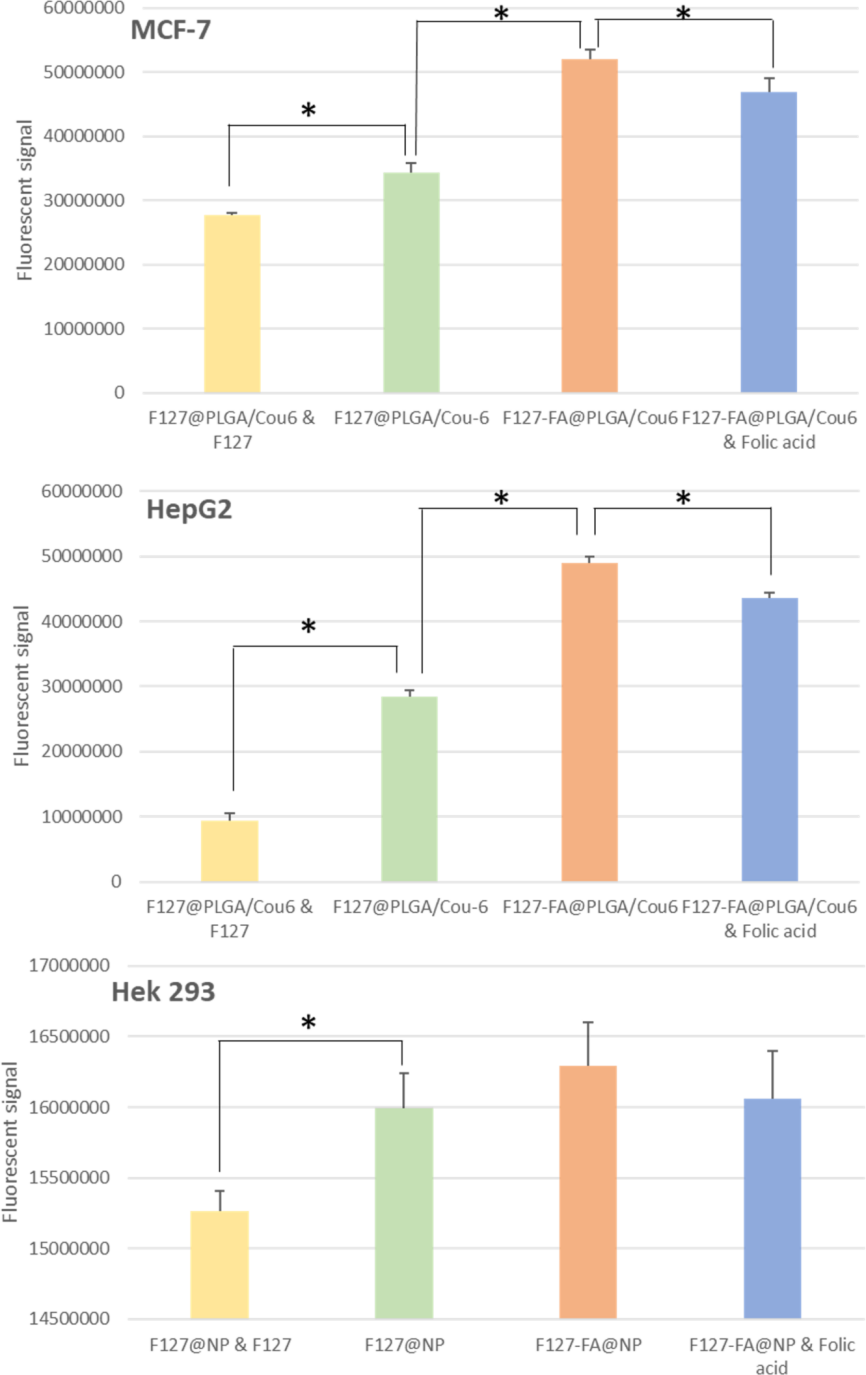
Uptake of F127@PLGA/Cou-6 and F127-folate@PLGA/Cou-6 in folate receptor-expressing cells (MCF-7, HepG2) and HEK 293 cells, which do not express the folate receptor. Cells represented in yellow and blue were treated with F127 and folic acid 30 min prior to, respectively, F127@PLGA and F127-FA@PLGA incubation. *p < 0.05, with a *t*-test, one tail distribution, and two samples having the same variation. *n* = 3, cell number = 20,000 cells/well. The excitation and emission wavelengths are 450 and 510 nm.

Cancer cells overexpress many receptors and markers for their growth; one of them is the folate receptor [35]. The folate receptor binds to folic acid and would then transfer it into the cells. Folic acid plays an important role in cancer cells; it takes part in cell proliferation, methylation for gene expression, DNA replication, oxidative stress, and DNA mutation. Many studies and cancer drugs, therefore, have used folic acid as a targeting ligand for the folate receptor, which increases the treatment efficacy [12–13]. In our study, two folate receptor-positive cell lines (human breast carcinoma MCF7 and human hepatoma HepG2) and a folate receptor-negative cell line (HEK 293) were used. To test how well the particles could target the cell lines, they were incubated with free folic acid, free F127, F127-folate@PLGA/Cou-6, and F127@PLGA/Cou-6. After normalizing to the background, the fluorescent signals of F127-folate@PLGA/Cou-6 were much stronger in MCF7 and HepG2 than those of F127@PLGA/Cou-6 (Figure 2). The result showed that F127-folate@PLGA/Cou-6 was better at targeting folate receptor-expressing cells than F127@PLGA/Cou-6. In contrast, in HEK 293 cells, both types of NPs exhibited similar fluorescent signals.

To prove the targeting of folate-modified nanoparticles to the cancer cells, the cancer cells were pretreated with folic acid (2 µM) for 30 min. The folic acid would initially bind to the folate receptor, compete, and reduce the uptake of folate-containing nanoparticles. Therefore, folate@PLGA/Cou-6 was taken up much less by MCF7 and HepG2 cells when folic acid was present (Figure 2). In contrast, in the folate receptor-negative cells (HEK 293), the addition of folic acid did not change the level of the cell signal.

F127 also assists in the accumulation of the nanoparticles in the cell via clathrin-mediated endocytosis and caveolae-mediated endocytosis [9,44]. In our study, F127 enhanced the internalization of the nanoparticles in MCF7, HepG2, and HEK 293 cells (Figure 2). However, when treating the cells with F127 polymer (2 mg/mL), the fluorescent signals were significantly reduced. The results proved that in the wells that were pretreated with F127 polymer, the polymer floated free in the cell culture medium, bound to the receptor, and competed with the binding of F127 nanoparticles. Clathrin-mediated endocytosis and caveolin-mediated endocytosis occur in most of the cells; therefore, the internalization of the nanoparticles to the cell would be unselective [45]. Thus, there were fluorescent signals in the cells, most of which were incubated with F127-folate@PLGA/Cou-6 and F127@PLGA/Cou-6. The results are also presented in the fluorescent images of MCF7 and HepG2 after being incubated with Cou-6-loaded nanoparticles (Supporting Information File 1, Figure S2).

### Drug loading and its effect on cancer cells

Theragnostics consists of diagnosis and treatment. The term is not new to nanoscience, but it is always preferable to create a drug carrier that serves both goals; that was also the aim of the nanoparticle design in our study. In our study, the imaging agent is the near-infrared fluorescent dye IR780, while the treatment medication is the chemotherapy drug CHL. IR780 exhibits fluorescence in the infrared region, which is suitable for pre-clinical applications [12–13]. CHL in cancer treatment attaches to the DNA double strands and prevents them from splitting, disrupting the division and proliferation of cancer cells [12–13]. Consequently, our research employed these two compounds to evaluate the efficacy of our nanoparticle design.

The drug loading in nanoparticles was measured by measuring the absorbance at 258 nm for CHL and 780 nm for IR780. The results indicate that the nanoparticles contained 0.5% CHL and 0.26% IR780 (Table 1).

**Figure 3:**
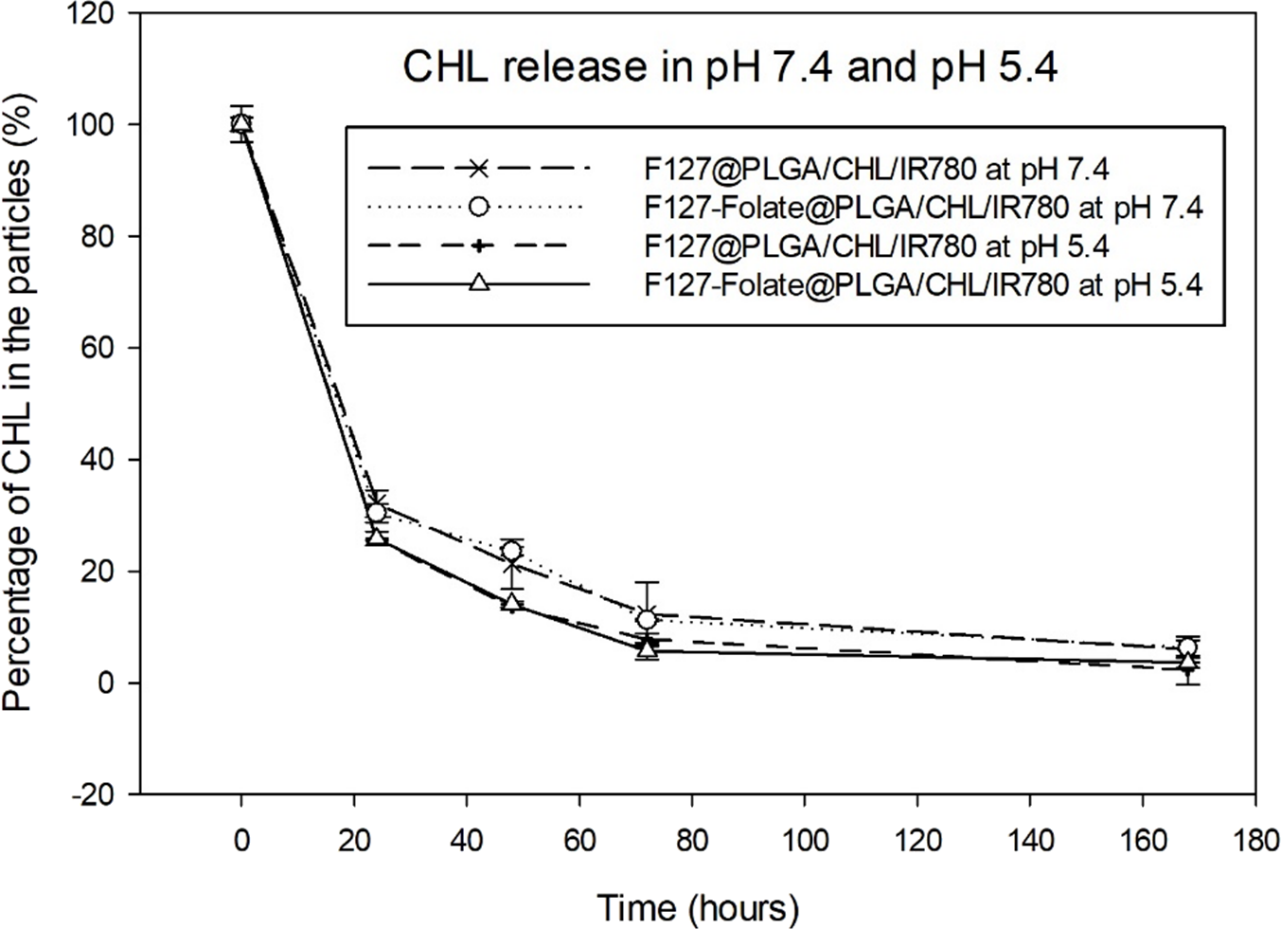
The release of chlorambucil from F127-folate@PLGA/CHL/IR780 and F127@PLGA/CHL/IR780 after 24, 48, 72, and 168 h of incubation in 0.1× PBS (NaCl 13.7 mM, KCl 0.27 mM, NaH_2_PO_4_ 1 mM, and KHPO_4_ 0.18 mM) at pH 7.4 and pH 5.4 at 37 °C; *n* = 3.

In order to mimic the conditions of drug release in vitro, the experiments were performed at 37 °C at pH 7.4 and pH 5.4. pH 7.4 represents the pH of physiological fluids in the body, while pH 5.4 is the pH value of the endosome. The CHL release was obtained by estimating the remaining CHL in the nanoparticles after incubation at 37 °C after 24, 48, 72, and 168 h at pH 7.4 and pH 5.4 in 0.1× PBS (NaCl 13.7 mM, KCl 0.27 mM, NaH_2_PO_4_ 1 mM, and KHPO_4_ 0.18 mM) (Figure 3). There were no differences between the CHL release from F127-folate@PLGA/CHL/IR780 and F127-folate@PLGA/CHL/IR780 at different time points at the same pH value. However, there were different concentrations of CHL in the nanoparticles at pH 7.4 and pH 5.4. After 24 h, the CHL content in the nanoparticles at pH 7.4 was about 32.2%, while at pH 5.4 it was 25.6%. The initial burst release of the CHL is explained by the drug’s loose attachment to the surface of the nanoparticles and the loosening of PLGA in the outer shell of the nanoparticles [3]. Notably, PLGA breaks down faster in an acidic environment; therefore, the medication is released more rapidly in the endosome of the cell than at a neutral pH [46]. There was a burst release of CHL at pH 5.4, which explained why the CHL in the nanoparticle in pH 5.4 media was lower than at pH 7.4. The CHL content in the nanoparticles continued to reduce to 22% after 48 h and 12.2% after 72 h in pH 7.4, while it decreased to 13.5% after 48 h and 4.5% after 72 h in pH 5.4. These numbers were significantly different (*p* < 0.05, *t*-test). After 168 h (7 days), the CHL content in the nanoparticle was about 3% to 6%.

**Figure 4:**
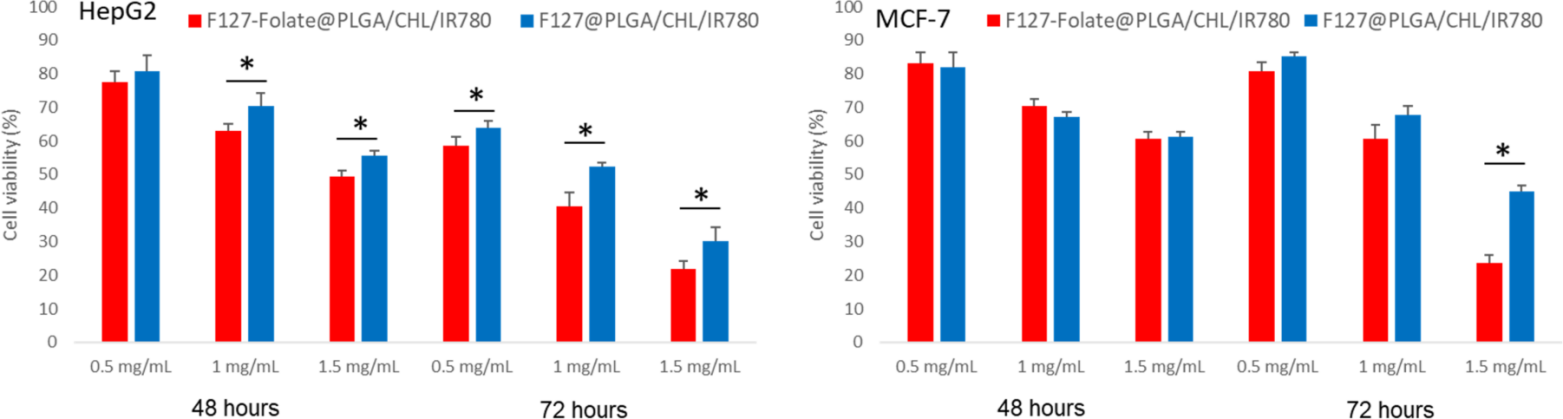
Cell viability of HepG2 and MCF-7. Control: cells received no treatment; the chlorambucil-treated cell at CHL concentrations of 6.57 μM, 13.14 μM, and 19.71 μM; F127-folate@PLGA/CHL/IR780; and F127@PLGA/CHL/IR780 at 0.5 mg/mL, 1 mg/mL, and 1.5 mg/mL. Cells were incubated for 48 h (red) and 72 h (blue). Initial cell inoculation: 1000 cells/well; *n* = 4, *p < 0.05.

The therapeutic efficacy of F127-folate@PLGA/CHL/IR780 and F127@PLGA/CHL/IR780 was evaluated regarding MCF7 and HepG2 cancer cells by MTT assay. The nanoparticles were incubated with the cells for 24, 48, and 72 h (Figure 4), with CHL serving as the positive control (Supporting Information File 1, Figure S3). For the first 24 h, the cell viability in every sample was greater than 90%. However, the cell viability decreased proportionately with the incubated concentration after 48 h, and it continued to decline up to 72 h. In the MCF7 test, the IC_50_ of F127-folate@PLGA/CHL/IR780 and F127@PLGA/CHL/IR780 were 1.04 mg/mL and 1.12 mg/mL, respectively, whereas the IC_50_ of the positive control CHL was 14.41 μM. HepG2 cell IC_50_ values for F127-folate@PLGA/CHL/IR780 and F127@PLGA/CHL/IR780 were 1.014 mg/mL and 1.1069 mg/mL, respectively, while the IC_50_ value for the positive control CHL was 13.57 μM.

When F127-folate@PLGA/CHL/IR780 was added to the HepG2 cells, the cell viability was much lower after 48 h than after incubation with F127@PLGA/CHL/IR780 at concentrations above 1 mg/mL. After 72 h, the cell viability after incubation with F127-folate@PLGA/CHL/IR780 was statistically lower than that of cells treated with F127@PLGA/CHL/IR780 (*p* < 0.05).

In MCF7 cells, there was no difference in cell viability after incubation with F127-folate@PLGA/CHL/IR780 and F127@PLGA/CHL/IR780 at concentrations of 0.5 and 1.0 mg/mL. However, after 72 h, the cell viability after treatment with F127-folate@PLGA/CHL/IR780 was significantly lower than that after treatment with F127@PLGA/CHL/IR780 at 1.5 mg/mL.

As demonstrated by the targeting evaluation experiment, the presence of folate on the surface of nanoparticles promotes the internalization of nanoparticles by cancer cells. Within the cell, the CHL released from the nanoparticles would bind to DNA and halt the development of cancer cells. Without CHL, the nanoparticles had no cytotoxic impact on the cells (data not shown). Previous research has demonstrated that the presence of folate on the particles increases the internalization of the nanoparticles into the cell [12,14,36–37].

Many studies have used F127 as a drug delivery agent because it is an FDA-approved material for use in the living body and assists the entry of nanoparticles into cells [9,47]. Cells treated with F127@PLGA/CHL/IR780 also showed toxicity of the NPs to the cancer cells, but not as high as F127-folate@PLGA/CHL/IR780. The toxicity of the nanoparticles could be due to the appearance of F127 on the surface of the nanoparticles, which assists it in entering the cell via clathrin-mediated endocytosis and caveolae-mediated pathways. Thus, our nanoparticles have the potential to serve as medication carriers. Besides, it could also be used as an imaging tracer because of the IR780 compound in the nanoparticles.

Many studies have encapsulated IR780 in nanoparticles to follow the targeting of nanoparticles as well as using it as a photosensitizer [23,48–52]. First, IR780 has an excitation wavelength of 777–780 nm and an emission wavelength of 798–823 nm, which is suitable for obtaining noninvasive near-infrared fluorescence images for clinical use. Second, IR780 generates heat when excited; therefore, it has been combined with other agents and used for photothermal therapy, triggering the release of drugs from nanoparticles for therapy [51–52]. Therefore, the appearance of IR780 in our nanoparticle in the study would bring some advantage in cancer treatment.

## Conclusion

In this work, we designed F127-folate@PLGA nanoparticles capable of carrying CHL and IR780. The formulation approach has produced nanoparticles of extremely homogeneous size. The F127-folate polymer on the surface of nanoparticles made it easier for nanoparticles to enter cells that express folate receptors, such as breast cancer cell MCF-7 and liver cancer cell HepG2. Cells that express folate negatively, like HEK 293, showed the same level of Cou-6 fluorescence. The F127-folate@PLGA/CHL/IR780 nanoparticles are more toxic to cancer cells than the F127@PLGA/CHL/IR780 nanoparticles, as shown by the MTT experiment. In conclusion, the proposed folate@PLGA/CHL/IR780 nanoparticles might be deemed a viable nanotechnology-based application technique for CHL and IR780 in theragnostic.

## Supporting Information

File 1 Additional figures regarding the NMR spectrum of F127-folate, the uptake of nanoparticle to the cancer cell, and the cell viability of CHL to cancer cells.

## Data Availability

The data that supports the findings of this study is available from the corresponding author upon reasonable request.
