# Supplementary material for: Therapeutic effect of F127-folate@PLGA/CHL/IR780 nanoparticles on folate receptor-expressing cancer cells
Source: Beilstein J Nanotechnol. 2024 Jul 31;15:954–64. doi: 10.3762/bjnano.15.78 (PMC11301043; doi:10.3762/bjnano.15.78)
Supplement: File 1 — Additional figures regarding the NMR spectrum of F127-folate, the uptake of nanoparticle to the cancer cell, and the cell viability of CHL to cancer cells. [file Beilstein_J_Nanotechnol-15-954-s001.pdf]

BEILSTEIN JOURNAL OF NANOTECHNOLOGY

# Supporting Information

for

# Therapeutic effect of F127-folate@PLGA/CHL/IR780 nanoparticles on folate receptor-expressing cancer cells

Thi Ngoc Han Pham, Phuong-Thao Dang-Luong, Hong-Phuc Nguyen, Loc Le-Tuan, Xuan Thang Cao, Thanh-Danh Nguyen, Vy Tran Anh and Hieu Vu_Quang

*Beilstein J. Nanotechnol.* **2024,** *15,* 954–964. doi:10.3762/bjnano.15.78

## Additional figures regarding the NMR spectrum of F127-folate, the uptake of nanoparticle to the cancer cell, and the cell viability of CHL to cancer cells

License and Terms: This is a supporting information file under the terms of the Creative Commons Attribution License (https://creativecommons.org/licenses/by/4.0). Please note that the reuse, redistribution and reproduction in particular requires that the author(s) and source are credited and that individual graphics may be subject to special legal provisions.
The license is subject to the *Beilstein Journal of Nanotechnology* terms and conditions: (https://www.beilstein-journals.org/bjnano/terms)

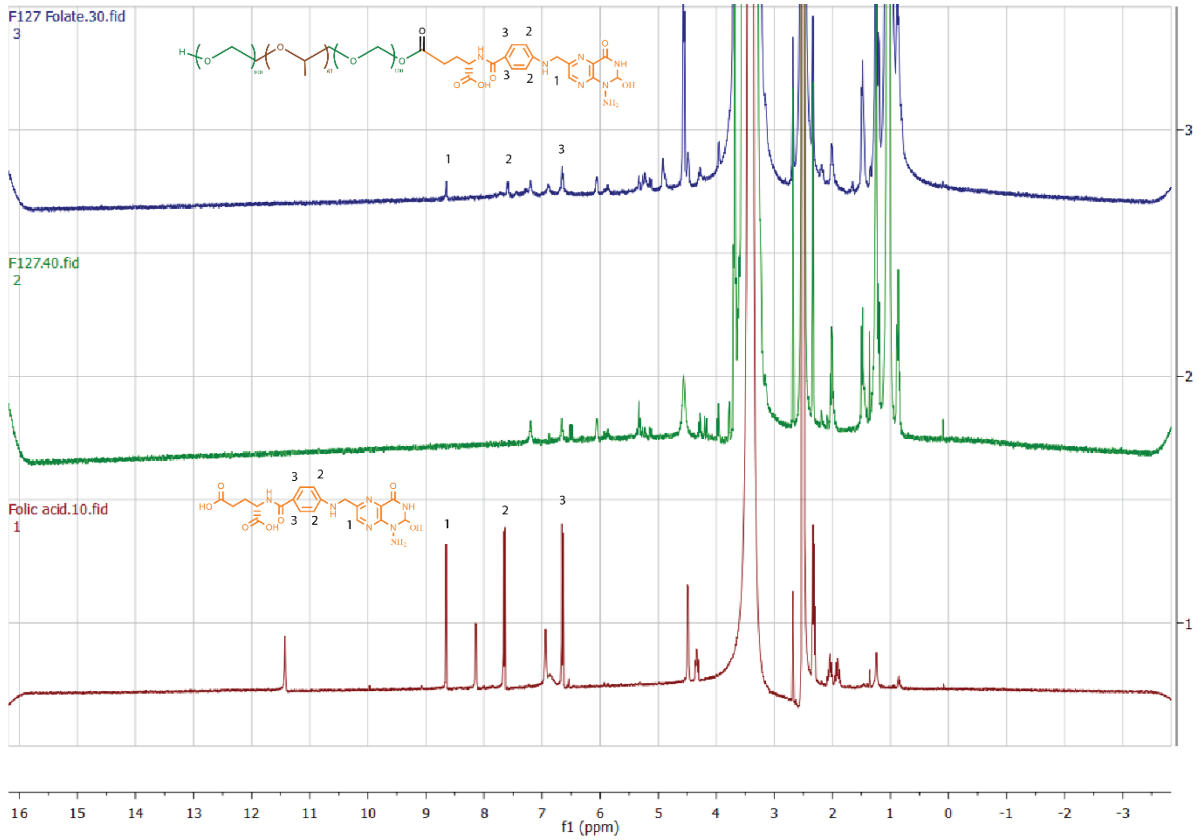


**Figure S1:** The $^{1}$H NMR spectrum of Folic acid, Pluronic F127 and c. The number show the position of proton in the spectrum.

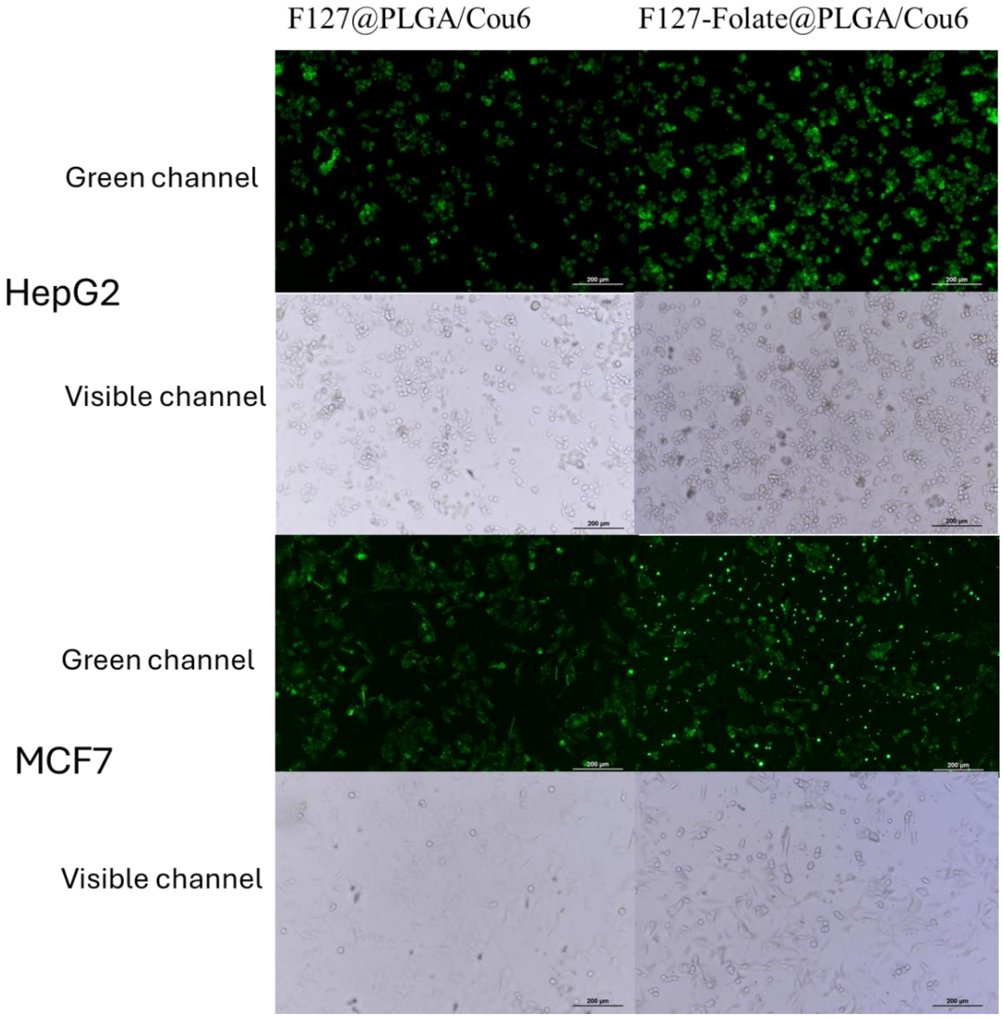


**Figure S2:** The uptake of F127@PLGA/Cou6 and F127-Folate@PLGACou6 to HepG2 and MCF7 after 3 hours of incubation.

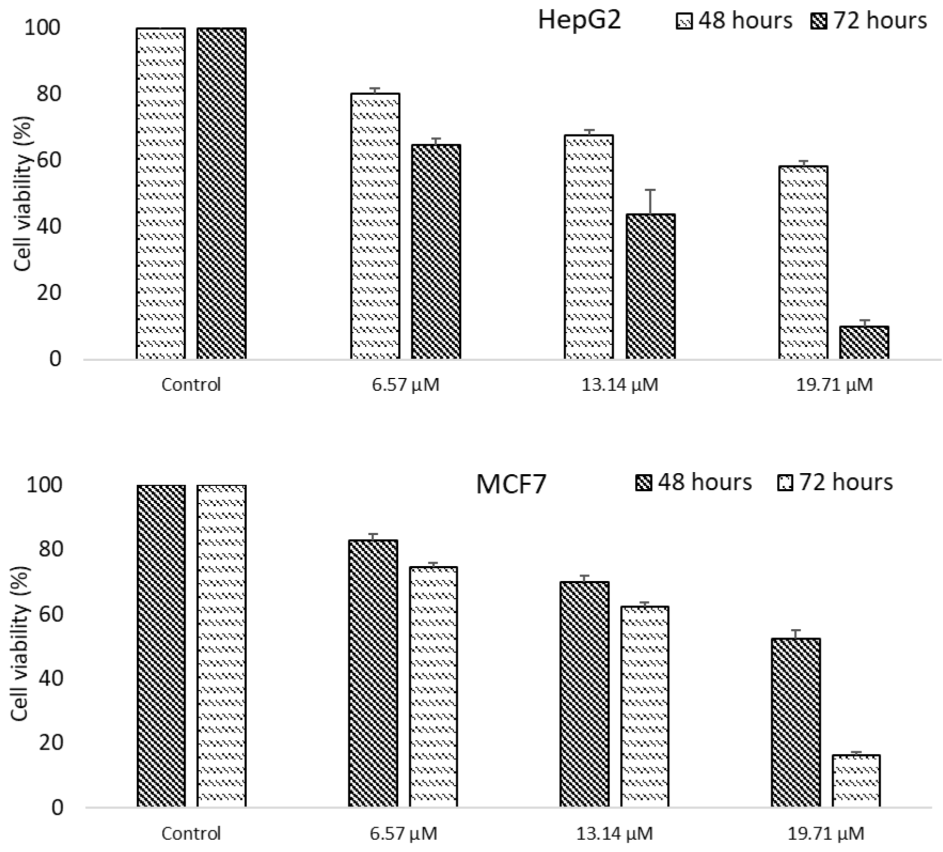


**Figure S3:** Cell viability of HepG2 and MCF7 after treating with chlorambucil at 6.57 µM, 13.14 µM and 19.71 µM in 48 hours and 72 hours.
